# Traumatic Brain Injury and Substance Related Disorder: A 10-Year Nationwide Cohort Study in Taiwan

**DOI:** 10.1155/2016/8030676

**Published:** 2016-09-27

**Authors:** Chieh-Hsin Wu, Tai-Hsin Tsai, Yu-Feng Su, Zi-Hao Zhang, Wei Liu, Ming-Kung Wu, Chih-Hui Chang, Keng-Liang Kuo, Ying-Yi Lu, Chih-Lung Lin

**Affiliations:** ^1^Graduate Institute of Medicine, College of Medicine, Kaohsiung Medical University, Kaohsiung 807, Taiwan; ^2^Department of Neurosurgery, Kaohsiung Medical University Hospital, Kaohsiung Medical University, Kaohsiung 807, Taiwan; ^3^Department of Neurosurgery, The No. 7 People's Hospital of Hebei Province, Dingzhou, Hebei 073000, China; ^4^Department of Neurosurgery, Yucheng People's Hospital, Yucheng, Shandong 251200, China; ^5^Department of Psychiatry, Kaohsiung Chang Gung Memorial Hospital and Chang Gung University College of Medicine, Kaohsiung 807, Taiwan; ^6^Department of Dermatology, Kaohsiung Veterans General Hospital, Kaohsiung 807, Taiwan; ^7^Cosmetic Applications and Management Department, Yuh-Ing Junior College of Health Care & Management, Kaohsiung 807, Taiwan; ^8^Department of Neurosurgery, Faculty of Medicine, College of Medicine, Kaohsiung Medical University, Kaohsiung 807, Taiwan

## Abstract

Whether traumatic brain injury (TBI) is causally related to substance related disorder (SRD) is still debatable, especially in persons with no history of mental disorders at the time of injury. This study analyzed data in the Taiwan National Health Insurance Research Database for 19,109 patients aged ≥18 years who had been diagnosed with TBI during 2000–2010. An additional 19,109 randomly selected age and gender matched patients without TBI (1 : 1 ratio) were enrolled in the control group. The relationship between TBI and SRD was estimated with Cox proportional hazard regression models. During the follow-up period, SRD developed in 340 patients in the TBI group and in 118 patients in the control group. After controlling for covariates, the overall incidence of SRD was 3.62-fold higher in the TBI group compared to the control group. Additionally, patients in the severe TBI subgroup were 9.01 times more likely to have SRD compared to controls. Notably, patients in the TBI group were prone to alcohol related disorders. The data in this study indicate that TBI is significantly associated with the subsequent risk of SRD. Physicians treating patients with TBI should be alert to this association to prevent the occurrence of adverse events.

## 1. Introduction

Traumatic brain injury (TBI) presents complex social problems and is a major cause of mortality and permanent disability in both developing and developed countries. In the US alone, an average of 1.4 million people experienced TBI annually. Although approximately 79% (1.1 million) of these are minor injuries that could be managed at emergency departments, approximately 17% (235,000) need to be treated in hospital, and as many as (4%) 50,000 are fatal [1, 2]. In Taiwan, the estimated annual average number of TBIs is 52,000, up to 25% of which are fatal [3]. Traumatic brain injury, also known as intracranial injury, occurs when an external force traumatically impacts the head in excess of the protective capacity of the cranium [4]. A TBI can cause chronic physical disability and neurobehavioral sequelae that produce highly disruptive cognitive and behavioural changes. Survivors often have difficulties maintaining personal relationships, and their work coping skills may be even more disabling than any residual physical disabilities [5]. Growing body of evidence also indicates that a history of TBI increases the risk of future brain injuries. Multiple brain injuries obviously have long lasting negative effects on mental health and some studies have reported a high prevalence of substance related disorder (SRD) in TBI patients [6–10]. However, an association between TBI and the subsequent development of SRD has not been well established [10–12]. That is, whether TBI itself increases SRD risk, especially in persons with no history of mental disorders at the time of injury, is unknown. Most studies of the connection between SRD and TBI have focused almost entirely on the causal relationships between the use or abuse of drugs or alcohol and TBI. Little population-based data for this association are available, and studies of the relationship between psychological problems, substance abuse, and TBI either have been contradictory or have used very small clinical samples [9, 10].

Large-scale studies of the relationship between TBI and subsequent SRD risk are rarely performed in population-based Asian cohorts. In 2011, Ilie et al. performed a telephone survey of a cross-sectional sample of 1999 Ontario, Canada, adults aged 18–93 years. Compared to subjects without a history of TBI, those with a history of TBI had higher adjusted odds of smoking and nonmedical use of cannabis and opioids. The TBI group also had higher odds of positive results in screenings for psychological distress. However, their survey results were potentially biased because they were based on self-reported data, and the response rate for the survey was only 51%. Additionally, they could not rule out the possibility that cognitive impairments affected the responses in the TBI group [13]. Therefore, this study retrospectively analyzed data from the Taiwan National Health Insurance Research Database (NHIRD) to clarify the relationship between TBI and subsequent risk of SRD.

## 2. Methods

### 2.1. Data Sources

The NHIRD used in this population-based cohort study comprises data for 99.9% of the 23.74 million residents of Taiwan and is maintained by the national healthcare system of Taiwan [3]. This retrospective cohort study analyzed 2000–2010 data contained in the Longitudinal Health Insurance Database (LHID). This database was developed by the Taiwan National Health Insurance Program and contains data for 1 million randomly selected patients. The LHID 2010 contains original claims data for 1 million beneficiaries randomly sampled during the period from January 1 to December 31, 2010. Distributions of gender, age, and insured payroll-related amounts do not significantly differ between the LHID 2010 and the original NHIRD. The large sample size of the dataset provides an opportunity to study SRD risk in patients. Diagnoses are coded according to the International Classification of Diseases, Clinical Modification, Ninth Revision (ICD-9-CM) code. This study was performed in accordance with the Declaration of Helsinki and was also evaluated and approved by the Institutional Review Board of Kaohsiung Medical University Hospital.

### 2.2. Subject Selection

This study analyzed 19,109 patients aged 18 years or older and diagnosed with TBI (ICD-9-CM codes 800, 803-804, and 850–854; operation codes 01.23, 01.24, 01.25, 0131, 01.39, and 02.01) during 2000–2010 [3]. To ensure accurate data, the TBI group was limited to patients who had received ≥2 TBI diagnoses during ambulatory visits or ≥1 diagnoses during inpatient care. The index date was defined as the date of the first clinical visit for TBI. Those diagnosed with any mental disorder (ICD-9-CM codes 290–319) before the index date were excluded from the TBI group. The TBI group was then divided into severe, moderate, and mild TBI subgroups. A severe TBI was defined as a TBI that received surgery during the course of inpatient treatment; a moderate TBI was defined as having hospitalized for TBI but not having undergone an operation; a mild TBI was defined as any history of head injury that did not receive inpatient treatment [14].

In this study, SRD was defined as a record of an ICD-9 code for SRD (ICD-9-CM codes 291, 292, 303.0, 303.9, 304, or 305) [15] entered by a psychiatrist and either ≥2 diagnoses of SRD in ambulatory visits or ≥1 diagnosis in inpatient care. The exclusion criterion was any diagnosis of SRD on or before the index date. SRD was further categorized as alcohol abuse (ICD-9-CM codes 291, 303.0, 303.9, and 305.0) [16] or illicit drug use (ICD-9-CM codes 292 and 304, and all 305 codes except 305.0) [16, 17].

**Figure 1:**
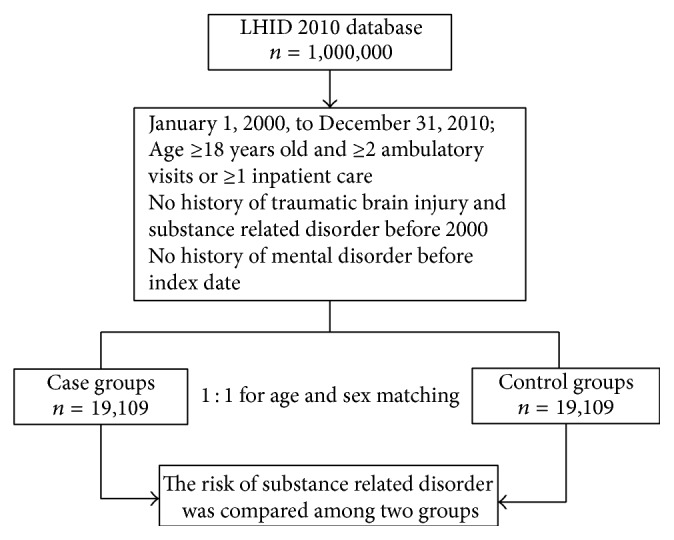
Flow diagram summarizing the process of enrollment. LHID: Longitudinal Health Insurance Database.

The non-TBI group was randomly selected from the registry of beneficiaries who had no TBI-related medical claims and no history of mental disorder. Each patient in the TBI group was matched with one person in the non-TBI group by age, gender, and year of TBI diagnosis (index year); thus, 19,109 patients were enrolled in the non-TBI group. A 1 : 1 ratio of TBI to non-TBI patients was maintained to enhance the power of statistical tests and to ensure a sufficient number of SRD cases for stratified analyses. A post hoc sample size calculation was performed to determine statistical power. Based on the event rate, the power for detecting a significant association between TBI and subsequent development of SRD exceeded 99% (*α* = 0.05).  Figure 1 shows a flowchart of the study procedure.

### 2.3. Outcome and Comorbidities

The outcome was the occurrence of SRD during the follow-up period. Both cohorts were followed up until December 31, 2010, or until a diagnosis of SRD.

Only a few clearly defined and modifiable risk factors have been defined for psychiatric disorders [18]. Some studies have used chronic obstructive pulmonary disease (COPD) as an indicator of smoking status and as a proxy for lifestyle-related behaviours [14, 19]. However, most studies have focused on largely unmodifiable factors such as age and gender. Potential confounders include many factors associated with both TBI and mental disorder, including common physical comorbidities such as hypertension (ICD-9-CM codes 401–405), diabetes mellitus (ICD-9-CM code 250), hyperlipidemia (ICD-9-CM code 272), coronary artery disease (ICD-9-CM codes 410–414), congestive heart failure (ICD-9-CM code 428), COPD (ICD-9-CM codes 491, 492, 494, and 496), malignancy (ICD-9-CM codes 140–208) [18], and unhealthy lifestyle behaviours [14]. The urbanization level and income-related insurance payment amounts were used to evaluate personal socioeconomic status. The urbanization level was categorized as urban, suburban, or rural [20]. The average monthly income was categorized into three groups: low (0–NT$20,000), medium (NT$20,000–40,000), or high (more than NT$40,000) [15, 21].

The Charlson Comorbidity Index (CCI) score was used to assess physical condition, that is, myocardial infarction, congestive heart failure, peripheral vascular disease, cerebrovascular disease, dementia, chronic pulmonary disease, rheumatic disease, peptic ulcer disease, mild and moderate or severe liver disease, diabetes (with or without chronic complication), hemiplegia or paraplegia, renal disease, any malignancy (including lymphoma and leukemia but excluding skin malignancy), metastatic solid tumor, and AIDS/HIV. The CCI scores were then categorized into four levels: 0, 1-2, 3-4, and ≥5. Each increase in the CCI score level corresponds with a stepwise increase in cumulative mortality [22].

### 2.4. Statistical Analyses

Chi-square test was used to compare distributions of categorical demographics and clinical characteristics between the TBI and non-TBI groups. Student's *t*-test and Wilcoxon rank-sum test were used as appropriate to compare mean age and follow-up time (*y*) between the two cohorts. The Kaplan-Meier analysis was used to estimate the cumulative incidence of SRD, and the differences between the curves were compared by 2-tailed log-rank test. In the TBI group, survival was calculated until hospitalization, an ambulatory visit for SRD, or the end of the study period (December 31, 2010), whichever came first. Incidence rates of SRD estimated in 1000 person-years were compared between the two cohorts. Cox proportional hazard regression models were used to calculate hazard ratios (HRs) and 95% confidence intervals (CIs) for SRD in the TBI group when the proportional hazards assumption was satisfied. Multivariable Cox models were adjusted for age, gender, income and urbanization level, CCI score, and relevant comorbidities. A 2-tailed *P* value of <0.05 was considered statistically significant. All data processing and statistical analyses were performed using Statistical Analysis Software, version 9.4 (SAS Institute, Cary, NC, USA).

## 3. Results

### 3.1. Baseline Characteristics of TBI and Non-TBI Groups

Table 1 compares the baseline demographic characteristics and comorbidities in the two cohorts. The mean age was 42.2 ± 17.6 years in the TBI group and 42.5 ± 17.2 years in the non-TBI group. In the TBI group, 56.98% were male. The percentages of patients with the following comorbidities were significantly higher in the TBI group compared to the non-TBI group, respectively: hypertension (36.67 versus 21.92, *P* < 0.001), diabetes mellitus (22.62 versus 12.99, *P* < 0.001), hyperlipidemia (31.06 versus 20.83, *P* < 0.001), coronary artery disease (6.69 versus 2.46, *P* < 0.001), congestive heart failure (7.04 versus 3.23, *P* < 0.001), COPD (24.41 versus 14.43, *P* < 0.001), and malignancy (8.46 versus 5.23, *P* < 0.001). The TBI group also had higher CCI scores. Moreover, patients in the TBI group were also more likely to qualify for insurance premium exemptions, less likely to pay high insurance premiums, and more likely to live in areas with low urbanization levels.

During the follow-up period, diagnoses of SRD significantly (*P* < 0.001) differed between the TBI group (1.78% (340 patients)) and the non-TBI group (0.62% (118 patients)). The TBI group also had significantly larger SRD subgroups for alcohol abuse (0.66 versus 0.13 in non-TBI, *P* < 0.001) and illicit drug abuse (0.87 versus 0.44 in non-TBI, *P* < 0.001).

### 3.2. Incidence and Risk of SRD

Table 2 stratifies the SRD incidence densities and HRs by gender, age, and comorbidity. During the follow-up period, 1.78% (340) patients in the TBI group and 0.62% (118) patients in the non-TBI group developed SRD. The overall SRD risk was 3.62 times greater in the TBI group compared to the non-TBI group (1.97 versus 0.41 per 1,000 person-years, resp.) after adjusting for age, gender, income, urbanization level, CCI, and related comorbidities (hypertension, diabetes mellitus, hyperlipidemia, coronary artery disease, congestive heart failure, COPD, and malignancy). The gender-specific analyses showed that, in both cohorts, the incidence of TBI was higher in men than in women (2.69 versus 0.57 per 1,000 person-years, resp., in the TBI group; 1.01 versus 0.19 per 1,000 person-years, resp., in the non-TBI group). Additionally, the SRD risk was significant in both men and women (adjusted HR = 3.69 versus 3.45, resp., *P* < 0.001).

The incidence of SRD was consistently higher in the TBI group at different ages, and the incidence rate consistently decreased with age. Additionally, the SRD risk decreased with age, and the age-specific risk analysis showed a significantly higher SRD risk in TBI patients aged younger than 40 years compared to those aged 40 years and older (HR = 5.03 versus 1.19; *P* for interaction <0.001). Regardless of comorbidities, SRD risk was higher in the TBI group than in the non-TBI group. The SRD risk contributed by TBI decreased in the presence of comorbidity (HR = 5.14 versus 2.59; *P* for interaction = 0.002).

**Figure 2:**
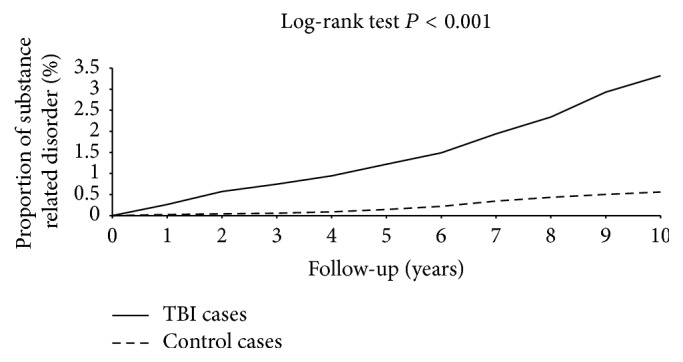
Cumulative incidence of substance related disorder among TBI (solid line) and control (dashed line) cases. TBI: traumatic brain injury.

Figure 2 compares the Kaplan-Meier curves for the cumulative incidence of SRD between the TBI and non-TBI groups at the 10-year follow-up. The cumulative incidence curves for SRD in the two cohorts showed a significantly higher incidence of SRD in the TBI group compared to the non-TBI group (log-rank test *P* < 0.001).

### 3.3. Predictors of SRD

The Cox regression analysis results for the TBI group revealed that the major risk factors for SRD were high CCI score (adjusted HR = 1.77; 95% CI = 1.57–2.00). Age and female gender had a protective role for SRD in these patients (Table 3).

### 3.4. SRD and TBI Severity

Table 4 shows that SRD risk increased with severity of TBI, particularly in those with severe TBI (adjusted HR = 9.01; 95% CI = 4.97–16.33).

### 3.5. Incidence and Risk of SRD Subtypes

Table 5 compares incidence rates and HRs for various outcomes between the TBI and non-TBI groups. After adjusting for covariates, the TBI group had a 6.22-fold higher incidence of alcohol abuse (95% CI = 3.94–9.84) and a 2.47-fold higher incidence of illicit drug use (95% CI = 1.83–3.32).

## 4. Discussion

To the best of our knowledge, this study is the first to perform a nationwide population-based analysis of the relationship between TBI and subsequent SRD in an Asian population. This study showed that SRD risk increases after TBI. SRD was identified in 1.78% (340) patients in the TBI group but in only 0.62% (118) patients in the non-TBI group. Overall, SRD risk was 3.62 times greater in the TBI group compared to the non-TBI group. In both genders, TBI was associated with a significantly increased risk of SRD. However, the incidence rate of post-TBI SRD decreased as age increased. Additionally, SRD risk increased with the severity of TBI. Specifically, compared to patients in the non-TBI group, SRD risk was 5.5 times higher in those with mild TBI and 6.5 times higher in those with moderate TBI. Comparisons of SRD subtypes in the TBI group further showed that alcohol abuse disorder was the leading one. Notably, patients in the TBI group were prone to suffer from alcohol abuse disorders.

Studies of the association between TBI and SRD have reported inconsistent results. A prospective survey of 939 health-maintenance organization members found that TBI survivors with no history of psychiatric disorder in the year prior to injury had an odds ratio of 4.5 for substance abuse within the 12 months after TBI. The odds ratio for substance abuse dropped to 1.4 at the third year after TBI. Prevalence rates for SRD increased from 7.3% pre-TBI to 14% at 1 year after TBI while SRD rates in matched non-TBI controls were 1.7% and 1.6% for the respective time periods [11]. Another study of 121 hospital inpatients with TBI and 133 controls by Ponsford et al. found that, in 25.4% of the TBI patients, hazardous levels of substance use were mentioned. Substance use increased and peaked by 2 years after injury. They concluded that substance use increased over time [23]. In contrast, an Australia study of sequential cohorts (aged 20–24, 40–44, and 60–64 years in wave 1) assessed TBI and SRD at baseline and 4 years later by using a survey methodology. Of the 7485 enrollees in the first wave of interviews, 89.7% were reinterviewed in the second wave of interviews. Between waves 1 and 2, 56 of the reported TBIs were mild (230.8/100000 person-years), and 44 were moderate (180.5/100000 person-years). The TBI risk was higher in males than in females and was highest in the cohort aged 20–24 years. Traffic accidents caused more moderate TBIs than mild TBIs. In wave 1 interviews, neither alcohol nor marijuana abuse is a predictor of TBI. In the second wave of interviews, TBI was not a predictor of substance related problems. However, the TBI incidence declined with age [10]. In Bombardier et al., changes in substance use from before to after TBI were investigated in 197 hospitalized adult patients. According to their data, a significant reduction in heavy drinking during the first year following TBI was reported [24].

The vast majority of TBI research has focused on the role of alcohol as a cause of TBI or risk factor for TBI rather than vice versa. However, the data analyzed in our study revealed that alcohol consumption increased after TBI and that alcohol use disorder was the most common SRD after TBI. Reports of the association between TBI and alcohol use disorder in the literature have been inconsistent. For example, a 2015 study found that a TBI group showed a higher incident rate ratio of developing alcohol use disorder (adjusted incidence rate ratio, 1.5) compared to the non-TBI group. The TBI group also had a higher risk of alcohol use disorder within 1 year after TBI [25]. In addition to these human studies, a rat model of TBI in Mayeux et al. showed that marked localized neuroinflammation at the TBI site was associated with post-TBI escalation of alcohol drinking [26]. In contrast, in Kreutzer et al., a comparison of self-reported alcohol use before and after TBI showed that, of the patients that were moderate-to-heavy drinkers before TBI, more than two-thirds of them reduced alcohol consumption, and approximately half of them quit drinking alcohol [27].

Although the exact mechanisms underlying the relationship between TBI and SRD are unclear, several possibilities could help explain the link between TBI and SRD. First, persistent inflammatory events caused by TBI in the nervous system result in gliosis, cerebral edema, and expression of proinflammatory cytokine. Notably, a large and growing body of literature indicates that the relationship between alcohol intake and inflammation is bidirectional; that is, alcohol causes inflammation of the brain, which induces an increase in alcohol consumption [28]. Therefore, TBI-related inflammatory events could induce an increase in alcohol intake. Thus, both TBI and long term alcohol intake promote neuroinflammatory events in the central nervous system. Alcohol intake after TBI was a kind of feed-forward mechanism wherein TBI-related inflammatory events promote alcohol intake, which further reinforces and amplifies inflammation in the nervous system. Second, substantial evidence indicates that another pathogenic cause of TBI is dysregulation of midbrain dopaminergic systems, which contributes to the chronic cognitive and behavioural sequelae associated with TBI [29]. Emerging evidence also indicates that TBI disrupts dopamine (DA) pathways. After electrical stimulation of the fore brain, experimental unilateral administration of controlled impacts to the parietal cortex of rats blunted striatal DA release and also decreased DA transporter [30, 31]. DA system hypofunction is also a major cause for the development of substance and alcohol use disorders [29, 32]. Furthermore, a frontal cortex TBI can cause an organic personality disorder tending to promote substance abuse [33–35]. TBI to the frontal cortex could also increase SRD risk by introducing long term executive cognitive deficits. The frontal lobes of the brain are involved in executive functions and also have roles in predicting consequences, decision making between actions, and suppressing unacceptable psychosocial responses [36]. Losing these functions is a key element of neurobiology of substance dependence and addiction [28].

The strength of this study is the use of a large representative population-based dataset to demonstrate an association between TBI and SRD risk. The large sample size also enabled analyses stratified by the time and the severity of TBI. However, several limitations of this study are noted. First, the diagnoses of TBI and SRD were based on ICD-9-CM codes entered in patient records. Therefore, one limitation of this study is the unknown accuracy of diagnostic codes entered in the database, which depends on the performance of clinical physicians. To correct for this limitation, only TBI diagnosed by surgeons and SRD diagnosed by psychiatrists where each had at least two consensus diagnoses were included in this study. Notably, the Taiwan Bureau of National Health Insurance regularly audits medical specialists to ensure the accuracy of their insurance claim codes. Therefore, doctors and medical institutions are motivated to enter diagnostic codes accurately because they are subject to large fines for coding errors. Additionally, the NHIRD has been used for many years in various studies [26–28]. A second limitation is that the TBI population is overrepresented by (often undiagnosed) substance abusers in particular, because alcohol and other illicit drugs so often are precipitating factors in the injury. So it remains possible that a large portion of the greater diagnosis is mediated by the eventual treatment seeking of patients that were substance users/abusers before injury. Third, the NHIRD does not contain details for some data that could compromise our findings, such as family history of mental disorder, marital status, personality characteristics, Glasgow Coma Scale, mechanism of trauma, and the duration of loss of consciousness. Fourth, most Taiwanese people are of Chinese ethnicity; further studies are needed to determine whether our findings are applicable in other ethnic groups. Another issue is that, despite the high coverage rate of the National Health Insurance system and the low payments for health care in Taiwan, people with SRD may not seek medical care because they prefer to avoid embarrassment or legal issues. Finally, since statistical significance may not indicate clinical significance, further clinical trials are needed to examine the underlying mechanisms of TBI and SRD and to confirm their relationship.

## 5. Conclusions

In conclusion, this nationwide population-based cohort study revealed that TBI increases the risk of subsequent SRD. However, further studies are needed to collect detailed data and to explore the mechanisms underlying this association. Timely interventions may help alleviate SRD associated with brain injury.

## Figures and Tables

**Table 1:** Baseline characteristics of patients with and without traumatic brain injury.

Variables	Traumatic brain injury		*P* value
	Yes (*N* = 19,109)	No (*N* = 19,109)	
*Mean age at enrollment (years, SD)*	42.2 (17.6)	42.5 (17.2)	0.088

*Age group, n (%)*			
18–39	9567 (50.07)	9567 (50.07)	
≥40	9542 (49.53)	9542 (49.53)	1.000

*Gender, n (%)*			
Men	10889 (56.98)	10889 (56.98)	
Women	8220 (43.02)	8220 (43.02)	1.000

*Income, n (%)*			
Low (<NT$20,000)	15624 (81.76)	15484 (81.03)	
Medium (NT$20,000–40,000)	2483 (12.99)	2330 (12.19)	
High (>NT$40,000)	1002 (5.24)	1295 (6.78)	<0.001

*Urbanization level, n (%)*			
Urban	11230 (58.77)	11612 (60.77)	
Suburban	6303 (32.98)	6296 (32.95)	
Rural	1576 (8.25)	1201 (6.28)	<0.001

*Charlson Comorbidity Index, n (%)*			
0	4542 (23.77)	7752 (40.57)	
1-2	7372 (38.58)	7411 (38.78)	
3-4	3760 (19.68)	2434 (12.74)	
≥5	3435 (17.98)	1512 (7.91)	<0.001

*Comorbidity, n (%)*			
Hypertension	7007 (36.67)	4189 (21.92)	<0.001
Diabetes mellitus	4323 (22.62)	2482 (12.99)	<0.001
Hyperlipidemia	5936 (31.06)	3980 (20.83)	<0.001
Coronary artery disease	1279 (6.69)	471 (2.46)	<0.001
Congestive heart failure	1345 (7.04)	618 (3.23)	<0.001
Chronic obstructive pulmonary disease	4664 (24.41)	2758 (14.43)	<0.001
Malignancy	1616 (8.46)	999 (5.23)	<0.001

*Newly diagnosed substance related disorders, n (%)*	340 (1.78)	118 (0.62)	<0.001
Alcohol abuse	127 (0.66)	25 (0.13)	<0.001
Illicit drug use	166 (0.87)	84 (0.44)	<0.001
Combined alcohol and illicit drug use	47 (0.25)	9 (0.05)	<0.001

*Mean age at diagnosis of substance related disorder (years, SD)*	33.5 (11.9)	41.0 (13.3)	<0.001

SD: standard deviation.

**Table 2:** Incidence and hazard ratios of substance related disorder by demographic characteristics and comorbidity among patients with or without TBI.

Variables	Patients with TBI		Patients without TBI		Compared to non-TBI		*P* for interaction
	Substance related disorder	Rate	Substance related disorder	Rate	Crude HR^a^ (95% CI)	Adjusted HR^a^ (95% CI)	
*Overall*	340	1.97	118	0.41	5.71 (4.58–7.12)^c^	3.62 (2.87–4.57)^c^	

*Gender*							
Men	265	2.69	94	0.57	5.64 (4.40–7.23)^c^	3.69 (2.84–4.79)^c^	
Women	75	1.01	24	0.19	5.88 (3.64–9.49)^c^	3.45 (2.06–5.78)^c^	

*Stratify by age*							
18–39	256	2.79	65	0.45	7.20 (5.44–9.53)^c^	5.03 (3.76–6.72)^c^	<0.001
≥40	84	1.04	53	0.37	3.39 (2.39–4.81)^c^	1.91 (1.33–2.75)^c^	

*Comorbidity* ^b^							
No	122	1.68	54	0.31	6.51 (4.69–9.04)^c^	5.14 (3.69–7.16)^c^	0.002
Yes	218	2.17	64	0.57	4.55 (3.42–6.06)^c^	2.59 (1.93–3.49)^c^	

Rate, incidence rate per 1000 person-years; 95% CI, 95% confidence interval; HR, hazard ratio; TBI, traumatic brain injury.

^a^Model adjusted for age, gender, income, urbanization level, Charlson Comorbidity Index, and relevant comorbidities (hypertension, diabetes mellitus, hyperlipidemia, coronary artery disease, congestive heart failure, chronic obstructive pulmonary disease, and malignancy).

^b^Patients with any examined comorbidities, including hypertension, diabetes mellitus, hyperlipidemia, coronary artery disease, congestive heart failure, chronic obstructive pulmonary disease, and malignancy, were classified as the comorbidity group.

^c^ *P* < 0.001.

**Table 3:** Cox regression model: significant predictors of substance related disorder after TBI.

Variables	Adjusted HR^a^	(95% CI)	*P* value
Charlson Comorbidity Index	1.77	(1.57–2.00)	<0.001
Age	0.54	(0.48–0.59)	<0.001
Female gender	0.38	(0.29–0.49)	<0.001

HR, hazard ratio; 95% CI, 95% confidence interval; TBI, traumatic brain injury.

^a^Model adjusted for age, gender, income, urbanization level, Charlson Comorbidity Index, and relevant comorbidities (hypertension, diabetes mellitus, hyperlipidemia, coronary artery disease, congestive heart failure, chronic obstructive pulmonary disease, and malignancy).

The adjusted HR and 95% CI were estimated by a stepwise Cox proportional hazards regression method.

**Table 4:** Incidence and hazard ratios for substance related disorder stratified by the severity of TBI.

Variables	*N*	Substance related disorder	Rate	Crude HR^a^ (95% CI)	Adjusted HR^a^ (95% CI)
Without TBI	19109	118	0.41	1.00 (reference)	1.00 (reference)
Mild TBI	5757	79	1.84	8.73 (6.25–12.19)^b^	5.50 (3.89–7.78)^b^
Moderate TBI	12786	245	1.97	9.23 (6.31–13.48)^b^	6.50 (4.41–9.58)^b^
Severe TBI	566	16	3.31	19.14 (10.75–34.05)^b^	9.01 (4.97–16.33)^b^

Rate, incidence rate per 1000 person-years; 95% CI, 95% confidence interval; HR, hazard ratio; TBI, traumatic brain injury.

^a^Model adjusted for age, gender, income, urbanization level, Charlson Comorbidity Index, and relevant comorbidities (hypertension, diabetes mellitus, hyperlipidemia, coronary artery disease, congestive heart failure, chronic obstructive pulmonary disease, and malignancy).

^b^ *P* < 0.001.

**Table 5:** Incidence rates and hazard ratios of different substance related disorder risk in patients of TBI compared those without TBI.

Variables	Patients with TBI		Patients without TBI		Compared to non-TBI	
	Event	Rate	Event	Rate	Crude HR^a^ (95% CI)	Adjusted HR^a^ (95% CI)
Overall substance related disorder	340	1.97	118	0.41	5.71 (4.58–7.12)^b^	3.62 (2.87–4.57)^b^
Alcohol abuse	127	0.73	25	0.09	8.72 (5.60–13.58)^b^	6.22 (3.94–9.84)^b^
Illicit drug use	166	0.96	84	0.29	4.19 (3.17–5.53)^b^	2.47 (1.83–3.32)^b^
Combined alcohol and illicit drug use	47	0.27	9	0.03	8.89 (4.29–18.43)^b^	5.19 (2.43–11.10)^b^

Rate, incidence rate per 1000 person-years; 95% CI, 95% confidence interval; HR, hazard ratio; TBI, traumatic brain injury.

^a^Model adjusted for age, gender, income, urbanization level, Charlson Comorbidity Index, and relevant comorbidities (hypertension, diabetes mellitus, hyperlipidemia, coronary artery disease, congestive heart failure, chronic obstructive pulmonary disease, and malignancy).

^b^ *P* < 0.001.
